# The Cognitive Model of Negative Symptoms in Schizophrenia: A Hierarchical Component Model With PLS-SEM

**DOI:** 10.3389/fpsyt.2021.707291

**Published:** 2021-07-22

**Authors:** Ali Ebrahimi, Hamid Poursharifi, Behrooz Dolatshahi, Omid Rezaee, Hamid Reza Hassanabadi, Farooq Naeem

**Affiliations:** ^1^Department of Clinical Psychology, University of Social Welfare and Rehabilitation Sciences, Tehran, Iran; ^2^Department of Psychiatry, University of Social Welfare and Rehabilitation Sciences, Tehran, Iran; ^3^Department of Clinical Psychology, Faculty of Psychology and Educational Sciences, Kharazmi University, Tehran, Iran; ^4^Department of Psychiatry, University of Toronto & Centre for Addiction and Mental Health, Toronto, ON, Canada

**Keywords:** negative symptom, schizophenia, cognitive model, structural equating modeling, hierarachical model

## Abstract

The cognitive model of negative symptoms suggests that some dysfunctional beliefs mediate the relationship between neurocognitive deficits and negative symptoms and disability. This study tested the hypothesis that dysfunctional performance beliefs mediate neurocognitive deficits, negative symptoms, and disability. We used a hierarchal component model with 85 men patients diagnosed with chronic schizophrenia. Results showed a moderate to strong correlation between dysfunctional performance beliefs, neurocognitive deficits, negative symptoms, and disability. These results support the Hierarchal component model (HCM) of the cognitive model of negative symptoms. Our results indicated that the disability in schizophrenia is mediated through dysfunctional performance beliefs, neurocognitive deficits, and negative symptoms pathway. Further, dysfunctional performance beliefs have a crucial role in this pathway. Therefore, targeting this vicious cycle of dysfunctional beliefs can improve disability in patients with schizophrenia.

## Introduction

Negative symptoms such as diminished emotional expression, avolition, alogia, anhedonia, and asociality account for significant disability in persons diagnosed with schizophrenia (1). Approximately 60% of people with schizophrenia suffer from negative symptoms that persist despite treatment (2, 3). The negative symptoms can be disabling and can significantly burden psychosocial health, occupational functioning, and quality of life in people with schizophrenia (4, 5). Psychotropic medications have limited efficacy on negative symptoms (6–8). Evidence indicates that psychotropic medications have little efficacy on the real-world functioning of people with schizophrenia in general (9, 10). Similarly, side effects of antipsychotic drugs might lead to secondary negative symptoms or at least exacerbate negative symptoms (3, 11, 12).

In addition to negative symptoms, cognitive deficits can be troubling features of schizophrenia, adding to their real-world functioning, with almost 98% of the persons with schizophrenia suffering from cognitive deficits (13, 14). Cognitive deficits such as processing speed, attention, vigilance, working memory, verbal learning, visual learning, reasoning, problem-solving, and social cognition are common cognitive deficits in persons with schizophrenia (15, 16).

The cognitive model of negative symptoms suggests that the dysfunctional beliefs such as pessimistic beliefs about performance (e.g., “*If I fail at my work, then I am a failure as a person”)* and need for approval (e.g., “*If someone disagrees with me, it probably indicates he does not like me”)* mediate the relationship between neurocognitive deficits, negative symptoms, and disability (17–20). Grant and Beck (20) suggest that the psychological aspects of negative symptoms have been less acknowledged. They suggested that the psychological reaction of patients to their neurocognitive deficits (e.g., dysfunctional beliefs) exacerbates negative symptoms and disability (20, 21).

Several studies have examined the cognitive model of negative symptoms. For example, Horan et al. (4) reported the association between dysfunctional beliefs and negative symptoms with quality of life in schizophrenia. However, this study has been criticized for not conducting an in-depth analysis of dysfunctional beliefs. In another study, Green (22) tested functional impairment in schizophrenia through a single-path model from early visual perception, social cognition, defeatist beliefs, and negative symptoms to functional outcomes. They found that defeatist beliefs and negative symptoms mediate the relationship between perception and functional outcomes. Quinlan et al. (23) examined the mediating role of dysfunctional beliefs in the relationship between neurocognitive deficits, negative symptoms, and functional outcomes in patients diagnosed with schizophrenia and schizoaffective disorders. Their result supported the mediating role of dysfunctional beliefs in the relationship between neurocognitive deficits and functional outcomes.

In a recent study, Luther et al. (24) tested the cognitive model of negative symptoms in a community sample. Their results showed a significant path from self-efficacy to negative symptoms and the mediating role of defeatist beliefs. Further, they found a direct relationship between defeatist beliefs and the negative symptoms.

Reviewing the literature of the cognitive model of negative symptoms [e.g., (4, 20, 24)] indicated that the previously proposed models consisted of simple path analysis. Conceptually, it is often better to use hierarchical component models rather than standard one-dimensional structures because their use often reduces the number of structural model relationships, making the PLS path model more parsimonious and easier to understand (25). For example, in most of the currently proposed models [e.g., (4, 20, 22–24)], it has not been well-explained that what type of dysfunctional beliefs is specific and more strongly related to negative symptoms (e.g., performance evaluation, need for approval subscale). Also, while measurement model (outer model) misspecifications is a threat to the validity of SEM results, earlier models seem to have ignored it (26). Therefore, a separate study is needed to examine the cognitive model of negative symptoms using the hierarchical component model (HCM).

Toward this end, the present study is the first one designed to examine the cognitive model of negative symptoms using the hierarchical component model (HCM). In the current study, we utilized the hierarchal component method (HCM) using the Partial Least Squares-Structural Equation Modeling (PLS-SEM). This method is a composite-based approach for modeling complicated interrelationships between observed and latent variables, which has become popular in recent years (27). In addition, PLS-SEM has several advantages over other methods such as first-generation and covariance-based SEM. For example, PLS-SEM is an exploratory method based on an ordinary least squares regression method that predicts the path relationships in complex models. Additionally, PLS-SEM does not require assumptions about the normal distribution of the data and works well with small sample sizes and complex models (25).

Furthermore, the present study implemented a comprehensive assessment battery, that is, neuropsychological tests based on the MATRICS Consensus Cognitive Battery (MCCB), an agreed-upon battery for assessing negative symptoms in schizophrenia (28). MCCB is a performance-based measurement method, and previous studies (4, 23) have recommended using such performance-based assessment tools instead of merely relying on self-report and clinician-rated measures, which improves the accuracy of the measurement that enhances the fitness of a model.

In the current study, we hypothesizeed that dysfunctional performance beliefs significantly mediate the relationship between neurocognitive deficits, negative symptoms, and disability hierarchically in a patient with schizophrenia. We also expected to find significant associations between neurocognitive deficits, dysfunctional performance beliefs, negative symptoms, and disability in patient with schizophrenia.

## Materials and Methods

### Participants

Participants included 100 male patients diagnosed with a schizophrenia spectrum disorder in Tehran Razi Psychiatric Center and were recruited through the purposive sampling method. Data from 15 patients were excluded from the study because of the patients' lack of cooperation and their incomplete data. Thus, we analyzed data from the remaining 85 participants using a structured form; information on the patients' primary demographic data, diagnosis, duration of illness, and psychotropic use, were recorded. Patients have been prescribed Second-generation antipsychotics, Antidepressants, Mood stabilizers, Concomitant medications, and did no patients receive first-generation antipsychotics (For demographics information, See Table 1). While the determination of appropriate sample size is a critical issue in SEM, there is no consensus in the literature regarding the appropriate method for estimating sample size for SEM. Notwithstanding, some evidence suggests that simple SEM models could be meaningfully tested even if the sample size is quite small (29, 30). Also, we used PLS-SEM for data analysis, which is not sensitive to small sample sizes (25). In the current study, inclusion criteria were: (a) being 20–60-year-old (b) at least 2 years duration of illness since the onset of schizophrenia spectrum disorder, (c) presence of significant negative symptoms with SANS scores above the cut-off point of 24, and (d) being able to read and write in the Persian language. Exclusion criteria included: (a) a brain injury, learning disability or physical disability, and neurological disease (e.g., Epilepsy, Alzheimer disease, Dementia, Parkinson disease, Multiple sclerosis) that interfere with the assessment process, (b) side effects of psychiatric medications that interfere with the assessment process (c) presence of acute psychotic symptoms (delusions and hallucinations) that were assessed by SCID-5 in the pre-assessment stage, and (d) being severely disturbed by substance use.

**Table 1:** Demographic characteristic.

	**Patients (*****N*** **=** **85)**			
	**Mean**	**SD**	***t***	**Sig**.
Age (year)	45.63	8.97	0.005	0.99
Education (year)	10.0	1.94	−122.88	0.00^**^
Length of condition (year)	13.0	1.68	−140.35	0.00^**^
**Diagnosis**	***N***	**%**		
**Schizophrenia**			
Multiple episodes in partial remission	14	16.5		
Multiple episodes in full remission	64	75.3		
**Schizoaffective**			
Multiple episodes in full remission	5	5.9		
Multiple episodes in partial remission	2	2.4		
**Medication**	***N***	**%**		
Second-generation antipsychotics	55	65		
Antidepressants	15	18		
Mood stabilizers	7	8		
Concomitant medications	8	9		

** *p < 0.001*.

### Assessments

#### Structured Clinical Interview for DSM-5 (SCID-5)

SCID-5 is a semi-structured clinical interview used to diagnose psychiatric disorders based on DSM-5 diagnostic criteria. This interview is designed to reduce interview-related problems, clinical errors, and clinical judgment. The Persian translation of SCID-5 has been found to have acceptable reliability and validity for various categorical diagnoses in different clinical settings (31, 32).

#### The Scale for the Assessment of Negative Symptoms (SANS)

The Scale for the Assessment of Negative Symptoms (SANS) includes 24 items and categorizes negative symptoms into five dimensions, including Blunted Affect, Alogia, Avolition and Apathy, Asociality, and Attention (33). The Persian version of SANS has an excellent internal consistency (α = 0.94), and test-retest reliability (*r* = 0.92) (34). In the current study, the internal consistency of SANS was in a good range (α = *0.8*2).

#### Dysfunctional Attitude Scale [DAS; Weissman and Beck (17)]

DAS consists of 40 items designed to measure underlying beliefs about depressive symptoms. Fifteen items of DAS assess dysfunctional beliefs about performance, and 10 items measure the need for approval subscale. The measure is completed based on a 7-point Likert scale from 1 = *strongly agree* to 7 = *strongly disagree*. The Persian version of DAS showed good test-retest reliability (*r* = 0.76) (35). In the present study, the internal consistency of DAS was in the excellent range (α = 0.82).

#### MATRICS Consensus Cognitive Battery (MCCB)

Measurement and treatment research to improve cognition in schizophrenia consensus cognitive battery (MCCB) is a standard cognitive assessment method in Schizophrenia. The MCCB measures Processing Speed, Attention/Vigilance, Working Memory, Verbal Learning, Visual Learning, Reasoning/Problem Solving, and Social Cognition. It has a high test-retest reliability (28). In the current study, the internal consistency of 0.75 was reported for MCCB.

#### World Health Organization Disability Assessment Schedule (WHODAS 2.0)

This 36-item self-administered questionnaire assesses disability in general areas of life. WHODAS 2.0 subscales include Cognition, Mobility, Getting Along, Life Activities, Participation, and Self-Care. The total Cronbach's alpha of 0.98 has been reported for the Persian version of WHODAS 2.0 total score, and scores of 0.97, 0.98, 0.98, 0.98, and 0.97 has been found for the general population, substance abusers, alcohol abuser sample, patients with mental disorders, and patients with the physical illness, respectively (36, 37). The internal consistency of WHODAS 2.0 was 0.80 in the current study.

### Procedure

The study received ethical approval from the Research Ethics Committee of the University of Social Welfare and Rehabilitation Sciences (IR.USWR.REC.1399.103). All participants were informed about the aims of the study and the confidentiality of the data. Those who provided written informed consent were invited to participate. Each assessment lasted between 3 and 5 h. Diagnostic assessments to confirm diagnosis criteria of schizophrenia spectrum disorder were carried out by a psychiatrist and a clinical psychologist using the Persian Version of Structured Clinical Interview for DSM-5, SANS, MCCB, DAS, and WHODAS 2.0. These assessments were carried out between July 2020 and November 2020.

### Analyses

Descriptive statistics and correlational analysis were performed using SPSS 22.0. To deal with outliers and missing data, the Boxplot method and the Series mean method was used. Finally, 85 valid data were found eligible for analyses. We first conducted the descriptive analyses for the study sample and the measures (see Tables 1, 2).

**Table 2:** Descriptive statistic of Variables (*n* = 85).

**Variables**	**Domains**	**Min**	**Max**	**Mean**	**SD**	**Kurtosis**	**Skewness**	***t***	***Sig*.**
NCD	Speed of processing	104	211	14.14	42.2	0.077	−0.141	50.90	0.00^**^
	Attention/Vigilance	29	143	98.59	14.55	0.655	1.000	−42.40	0.00^**^
	Working memory	71	19	7.41	3.01	1.596	2.416	55.32	0.00^**^
	Verbal learning	37	74	49.74	10.21	0.332	−0.719	0.003	0.99
	Visual learning	31	79	49.09	9.58	0.551	0.657	0.006	0.99
	Reasoning/Problem solving	32	74	49.44	9.98	0.565	−0.432	0.001	0.99
	Social cognition	18	69	49.64	9.98	−0.481	0.353	0.006	0.99
	Total composite score	360	615	495.14	57.86	−0.314	−0.419	0.00	1.00
DAS	Performance evaluation	36	95	61.97	12.36	0.394	−0.009	0.003	0.99
	Need for approval	7	34	21.32	5.35	0.280	0.761	0.001	0.99
	Total	51	123	83.29	15.07	0.180	−0.208	0.003	0.99
NS	Blunted affect	0	35	10.97	8.19	1.218	0.723	0.005	0.99
	Alogia	0	25	8.57	6.51	0.983	−0.104	0.009	0.99
	Avolition and apathy	0	19	8.35	5.62	0.346	−1.365	0.014	0.98
	Asociality	0	23	7.06	5.05	0.874	0.404	0.871	0.387
	Attention	0	12	4.98	3.26	0.535	−0.532	0.019	0.98
	Total	0	95	40.50	25.69	0.649	−0.672	0.00	1.00
Dis	Cognition	6	27	14.79	6.28	0.266	−0.999	0.007	0.99
	Mobility	5	25	16.75	6.55	−0.349	−1.099	0.009	0.99
	Getting along	5	21	12.11	4.31	0.164	−0.548	14.48	0.00^**^
	Life activities	8	40	20.97	9.84	0.396	−1.192	3.72	0.00^**^
	Participation	8	37	19.61	9.12	0.173	−1.389	0.005	0.99
	Self–care	4	20	16.82	2.87	−1.252	3.631	0.002	0.99
	Total	36	156	101.07	34.51	0.107	−1.382	0.002	0.99

** *p < 0.00; NCD, Neurocognitive deficits; DPBs, Dysfunctional Performance Beliefs; NS, Negative Symptoms; Dis, Disability*.

Structural equation modeling (SEM) was conducted by Smart PLS 2.0.M3 (38). We performed a partial least squares—structural equation modeling method because PLS-SEM predicts path relationships in complex models more effectively. Also, data distribution criteria are not among PLS-SEM assumptions, and it applies efficiently with small sample sizes and more complex models (25). It is noteworthy that in comparison or other SEM approaches, the model fit indices in PLS-SEM are determined by *R*^2^ (explained variance), *T*-values, and beta paths (β) (25).

To execute PLS-SEM following steps were performed. First, we addressed preliminary considerations, such as data distribution assumption and multicollinearity. Second, we estimated the loadings, Cronbach's alpha, composite reliability (CR), average variance extracted (AVE), and *R*^2^ (explained variance) value for all variables (see Table 3). The visual learning subscale of neurocognitive deficits was removed because of the low loading factor (<3). We considered a factor loading of <3 representing a weak relationship, CR >0.7, and AVE >0.5 as was deemed to be desirable (39). Discriminant validity was also calculated to evaluate the measurement model. Discriminant validity indicates how the observed indicators are related to their constructs (25). Cross-loading estimation revealed that the correlation values for selected observed indicators were higher than other constructs. Therefore, each indicator showed the highest correlation with its construct and had the lowest correlation with other constructs.

**Table 3:** Assessment of measurement model of latent Variables (*n* = 85).

**Variables**	**Domains**	**Loading**	**CR**	**AVE**	**Cronbach's Alpha**	***R^**2**^***
NCD	Speed of processing	0.71				
	Attention/Vigilance	0.55				
	Working memory	0.30				
	Verbal learning	0.68				
	Reasoning/Problem solving	0.77				
	Social cognition	0.65				
	Total composite score		0.79	0.40	0.75	
DPBs	Performance evaluation	0.66				
	Need for approval	0.92				
	Total		0.77	0.64	0.82	0.07
NS	Blunted affect	0.84				
	Alogia	0.84				
	Avolition and apathy	0.92				
	Asociality	0.89				
	Attention	0.91				
	Total		0.94	0.78	0.82	0.56
Dis	Cognition	0.92				
	Mobility	0.87				
	Getting along	0.82				
	Life activities	0.92				
	Participation	0.90				
	Self–care	0.67				
	Total	0.92	0.94	0.73	0.80	0.74

*NCD, Neurocognitive deficits; DPBs, Dysfunctional Performance Beliefs; NS, Negative Symptoms; Dis, Disability; CR, Composite Reliability; AVE, Average variance extracted*.

**Figure 1:**
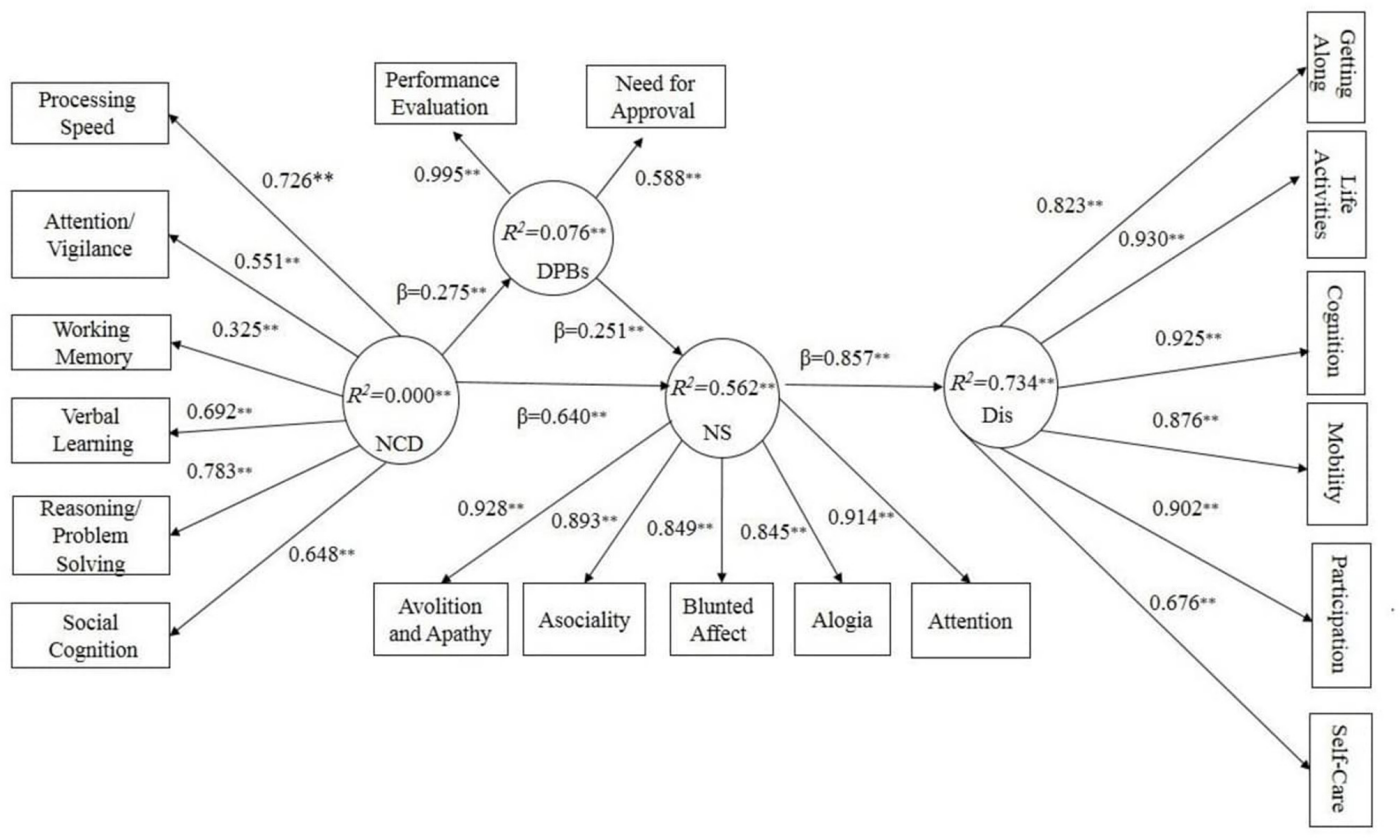
Structural model results show Dysfunctional Performance Beliefs mediate Neurocognitive deficits Negative Symptoms and disability. *NCD*, Neurocognitive deficits; *DPBs*, Dysfunctional Performance Beliefs; *NS*, Negative Symptoms; *Dis*, Disability. β, path coefficients; *R*^2^, explained variance; **p< *0.01*.

We also examined the discriminant validity of the latent variables using the Pearson correlation coefficient (see Table 4). Furthermore, to evaluate the overall measurement model fitness, we obtained the goodness-of-fit-index (GOF) measure, which was 0.54, indicating a strong model fit. Tenenhaus et al. (40) considered values of 0.01, 0.25, and 0.36 as weak, medium to high, and robust values for GOF. Then, after examining the measurement model, we performed PLS-SEM (see Figure 1), and the Sobel test was performed to assess indirect effects (41).

**Table 4:** Pearson correlations between neurocognitive deficits, dysfunctional performance believe, negative symptoms, and disability (*n* = 85).

	**Variables**	**1**	**2**	**3**	**4**
1	NCD	1			
2	DPBs	0.150^*^	1		
3	NS	0.510^**^	0.418^**^	1	
4	Dis	0.410^**^	0.403^**^	0.845^**^	1

*p < 0.01;*

* *p < 0.05; NCD, Neurocognitive deficits; DPBs, Dysfunctional Performance Beliefs; NS, Negative Symptoms; Dis, Disability*.

## Results

The Pearson correlation results indicated a significant positive association between neurocognitive deficits, dysfunctional performance beliefs, negative symptoms, and disability. All correlations were positively significant at the range of (*0.15* ≤ *r* ≤ *0.84; p*< *0.01, p*< *0.05*). The results showed that neurocognitive deficits are significantly correlated with dysfunctional performance beliefs (*r* = *0.150, p* = *0.05*), negative symptoms (*r* = *0.510, p* = *0.01*), and disability (*r* = *0.410, p* = *0.01*) (For full information, see Table 4).

### Structural Model

We started with a theoretical model based on our hypothesis that dysfunctional performance beliefs would mediate the association between neurocognitive deficits and negative symptoms with disability hierarchically. The results showed non-significant direct paths from neurocognitive deficits and dysfunctional performance beliefs to disability *(T* = *1.17*, β = *0.10; T* = *0.86 and* β= *0.05* respectively). By removing non-significant paths, our hypothesized model yielded a proper fit. As Figure 1 shows, neurocognitive deficits, as an exogenous construct, affect dysfunctional performance beliefs and negative symptoms significantly (*T* = *2.78*, β = *0.27, R*^2^ = *0.076, p*< *0.01*). Furthermore, neurocognitive deficits significantly affect negative symptoms as the dependent variable (*T* = *12.06* β= *0.64, p*< *0.01*). On the other hand, dysfunctional performance beliefs significantly mediated the association between neurocognitive deficits and negative symptoms (*T* = *3.48*, β= *0.23 R*^2^ = *0.562, p*< *0.01*). Finally, negative symptoms affected disability significantly (*T* = *9.54*, β = *0.85, R*^2^ = *0.734, p*< *0.01*). We assumed *T*-values above 1.96 as significant (25).

### Assessing the Indirect Effect in the Structural Model

Due to its parametric nature and reliance on unstandardized path coefficients, the indirect is not applicable in a PLS-SEM context (25). Therefore, the Sobel test was performed to assess the significance of the model's indirect effects. As Table 5 shows, the path from neurocognitive deficits to negative symptoms is mediated significantly by dysfunctional performance beliefs (*T* = *2.007, p*= *0.044)*. Similarly, the path from neurocognitive deficits to disability was mediated considerably by negative symptoms (*T* = *7.873, p* = *0.001)*. Also, a path from dysfunctional performance beliefs to disability was mediated significantly by negative symptoms (*T* = *2.856, p* = *0.004)*. Finally, dysfunctional performance beliefs did not significantly mediate the path from neurocognitive deficits to disability (*T* = *0.677, p* = *0.49)*.

**Table 5:** Sobel test results for indirect effects of neurocognitive deficits, dysfunctional performance beliefs, negative symptoms, and disability (*n* = 85).

**Independent**	**Mediating**	**Dependent**	***T-values***	***Std. error***	***p-value***
**variables**	**variables**	**variables**			
NCD	DPBs	NS	2.00	0.03	0.04^**^
NCD	DPBs	Dis	0.67	0.02	0.49
NCD	NS	Dis	7.87	0.06	0.001^**^
DPBs	NS	Dis	2.85	0.06	0.004^**^

*NCD, Neurocognitive deficits; DPBs, Dysfunctional Performance Beliefs; NS, Negative Symptoms; Dis, Disability; Std. Error, Standard Error.*

** *p < 0.01*.

## Discussion

To the best of our knowledge, this study is the first study that utilized the hierarchal component method (HCM) with a partial least squares—structural equation modeling (PLS-SEM) to examine that dysfunctional performance beliefs would mediate the association between neurocognitive deficits and negative symptoms with disability hierarchically in a patient with schizophrenia. Our results indicated that dysfunctional performance beliefs significantly mediated the association between neurocognitive deficits, negative symptoms, and disability hierarchically. In addition, a moderate to strong correlation was found between dysfunctional performance beliefs, neurocognitive deficits, negative symptoms, and disability. More specifically, dysfunctional performance beliefs had a moderate correlation with neurocognitive deficits and a strong correlation with negative symptoms and disability. Also, the highest correlation was found between disability and neurocognitive deficits. These findings are consistent with previous studies [e.g., (4, 20, 22–24, 42)].

Our results supported the hierarchal component model (HCM) of the cognitive model of negative symptoms. A growing body of studies proposed the dual-path (20), simple (4, 24), and structural (22, 23) models of the cognitive model of negative symptoms. The closest model to our suggested model proposed by Quinlan et al. (23) is a dual-path model with two mediational paths between neurocognition and real-world functioning, including one well-replicated pathway from neurocognition to functional skill capacity to real-world functioning, and the second from neurocognition to defeatist attitudes to negative symptoms to real-world functioning. However, our research differs from Quinlan et al.'s (23) study in several areas. First, the main difference between the current study and Quinlan et al.'s (23) is that we used the hierarchal component method (HCM) with PLS-SEM. This method offers a detailed and more accurate indicator. For example, in Quinlan et al.'s (23) suggested model, defeatist attitudes and functional capacity each affected the real-world functioning in one pathway, and it doesn't appear to be well-integrated and parsimonious. While in the original cognitive model of negative symptoms (20), the main emphasis is on dysfunctional beliefs and how they can lead to negative symptoms and disability, Quinlan et al. (23) introduced two pathways in which the role of defeatist attitude was not considered appropriately. Also, the subscales of defeatist attitudes, negative symptoms, and real-world functioning were not assessed. However, in our hierarchal component method (HCM), we assessed neurocognitive deficits, dysfunctional performance beliefs, negative symptoms, disability subscales; in addition, paths from neurocognitive deficits to dysfunctional performance beliefs to negative symptoms explained 73 percent of disability in Schizophrenia, making our model more detailed. However, it should be emphasized that because of different analysis approaches used in our research and Quinlan et al. (23), different indices were considered for examining model fitness. For example, we relied on *R*^2^ (explained variance), T-values, and beta paths (β) to examine model fitness, while Quinlan et al., (23) considered χ^2^, CFI, and RMSEA as model fit indices, which makes it difficult to compare the two models. Our findings (based on theoretical reasoning) revealed a more precise and detailed model of the cognitive model of negative symptoms. It means that, conceptually, disability in schizophrenia is affected by neurocognitive deficits, dysfunctional performance beliefs, and negative symptoms. Further, while each of these paths provides a weak and incomplete prediction of disability separately and directly, the indirect paths from neurocognitive deficits → dysfunctional performance beliefs → and negative symptoms better explain the disability in schizophrenia (see Figure 1, Table 5).

To conceptualize psychosocial mechanism underlying negative symptoms and disability in schizophrenia, our findings provide some evidence that neurocognitive deficits in schizophrenia can lead to failure experiences or failure expectations, which affect persons daily life functioning, leading to dysfunctional, and asocial attitudes and negative evaluation of their self and potentials (e.g., “*If I do not do well all the time, people will not respect m*e” or “*If I fail partly, it is as bad as being a complete failure”*) (17). Dysfunctional and asocial attitudes could lead to negative symptoms (e.g., apathy, indifference, withdrawing social relationships, a lack of engagement in purposeful actions) and interfere with their most social competencies. As a result, patients develop a dysfunctional attitude as defective mechanisms, which lead to repeated failure experiences, underestimating themselves, and low expectation of pleasurable experiences. Usually, this vicious cycle continues and is repeated constantly.

Our model supports the idea that negative symptoms serve as a maladaptive mechanism that protects individuals from the anticipated pain and rejection associated with engagement in constructive activity. Furthermore, beliefs induced by the stigma of mental illness (e.g., “*I won't be able to achieve anything or have meaningful relationships because I have schizophrenia*”) exacerbate the situation. Further, neurocognitive deficits can put the patient in a recurring cycle of frustration and failure, such as inaccurate goal setting and reduced ability to learn from errors (18, 43, 44).

The therapeutic implication of our results is that if patients with schizophrenia receive effective therapy to modify and disconfirm their dysfunctional beliefs, their daily life performance could significantly improve. In this context, different evidence-based versions of cognitive-behavioral therapy and cognitive remediation have emerged to target these issues s (9, 45–56).

There are several limitations to this study that need to be explained. First, despite using accurate assessment measures, we used a self-report tool (e.g., DAS), so it is recommended that future studies use more precise assessment tools, especially in measuring dysfunctional beliefs. Furthermore, our research design was a cross-sectional study, which does not confirm causal relationships; therefore, future research should focus on longitudinal studies. Similarly, this model can be tested with persons at different stages of the illness; in our study, we conducted on patients with chronic illness and predominantly negative symptoms. Also, Participants were prescribed second-generation antipsychotics, antidepressants, mood stabilizers, and Concomitant medications. Negative symptoms can be primary expressions of illness or secondary to other factors (e.g., depression, medication). To what degree the negative symptoms were primary or secondary cannot be estimated. In addition, side effects were not assessed systematically using a validated scale, only the classification of psychotropic drugs was recorded, and no information related to dosage was recorded.

Further, in the current study, we assessed positive symptoms using SCID-5 criteria; we recommend that future studies use valid measures such as the Scale for the Assessment of Positive Symptoms (SAPS) and other valid and reliable tools for assessing positive symptoms. Also, we did not measure the level of depressive symptoms, which is an essential source for secondary negative symptoms and should be included and controlled. Finally, the present study's sample included only men, so one should be careful not to generalize the results from this sample to other groups. Therefore, It is recommended that future studies include and study women and adolescents samples with schizophrenia spectrum disorder.

## Data Availability Statement

The raw data supporting the conclusions of this article will be made available by the authors, without undue reservation.

## Ethics Statement

The study received ethical approval from the Research Ethics Committee of the University of Social Welfare and Rehabilitation Sciences (IR.USWR.REC.1399.103). The patients/participants provided their written informed consent to participate in this study.

## Author's Note

The manuscript was extracted from a Ph.D. dissertation of the first author of the study conducted in the Department of Clinical Psychology, University of Social Welfare and Rehabilitation Sciences of Tehran, Iran.

## Author Contributions

AE designed the study and investigation and prepared the manuscript. HP, BD, OR, and HH supervised and reviewed the manuscript. FN review and editing the manuscript. All authors contributed to the article and approved the submitted version.

## Conflict of Interest

The authors declare that the research was conducted in the absence of any commercial or financial relationships that could be construed as a potential conflict of interest.

## Publisher's Note

All claims expressed in this article are solely those of the authors and do not necessarily represent those of their affiliated organizations, or those of the publisher, the editors and the reviewers. Any product that may be evaluated in this article, or claim that may be made by its manufacturer, is not guaranteed or endorsed by the publisher.
